# SIRT3-KLF15 signaling ameliorates kidney injury induced by hypertension

**DOI:** 10.18632/oncotarget.17165

**Published:** 2017-04-17

**Authors:** Na Li, Jie Zhang, Xuefang Yan, Chen Zhang, Hui Liu, Xiaolan Shan, Jingyuan Li, Yi Yang, Chengmin Huang, Peng Zhang, Yun Zhang, Peili Bu

**Affiliations:** ^1^ The Key Laboratory of Cardiovascular Remodeling and Function Research, Chinese Ministry of Education and Chinese Ministry of Health, The State and Shandong Province Joint Key Laboratory of Translational Cardiovascular Medicine, Qilu Hospital of Shandong University, Jinan, Shandong, China

**Keywords:** SIRT3, KLF15, honokiol, hypertensive kidney injury, podocyte

## Abstract

Renal fibrosis participates in the progression of hypertension-induced kidney injury. The effect of SIRT3, a member of the NAD^+^-dependent deacetylase family, in hypertensive nephropathy remains unclear. In this study, we found that SIRT3 was reduced after angiotensin II (AngII) treatment both *in vivo* and *in vitro*. Furthermore, SIRT3-knockout mice aggravated hypertension-induced renal dysfunction and renal fibrosis via chronic AngII infusion (2000 ng/kg per minute for 42 days). On the contrary, SIRT3-overexpression mice attenuated AngII-induced kidney injury compared with wild-type mice. Remarkably, a co-localization of SIRT3 and KLF15, a kidney-enriched nuclear transcription factor, led to SIRT3 directly deacetylating KLF15, followed by decreased expression of fibronectin and collagen type IV in cultured MPC-5 podocytes. In addition, honokiol (HKL), a major bioactive compound isolated from Magnolia officinalis (Houpo), suppressed AngII-induced renal fibrosis through activating SIRT3-KLF15 signaling. Taken together, our findings implicate that a novel SIRT3-KLF15 signaling may prevent kidney injury from hypertension and HKL can act as a SIRT3-KLF15 signaling activator to protect against hypertensive nephropathy.

## INTRODUCTION

Chronic kidney disease (CKD) is a severe complication of hypertension [1–3]. Glomerulosclerosis, renal tubule interstitial fibrosis and proteinuria are the main pathological characteristics of the hypertension-induced kidney injury [4–6]. Podocytes, as the terminally differentiated cells of glomerular filtration barrier, play a key role in maintenance of the glomerular filtration barrier so as to prevent proteinuria in CKD [7, 8]. In addition, podocytes damage or loss can result in renal fibrosis and CKD due to the accumulation of fibrosis factors [9]. Reducing fibrosis factors in kidneys, such as fibronectin and collagen type IV, could alleviate and even reverse renal fibrosis in hypertensive nephropathy.

SIRT3, a member of the evolutionary conserved family of NAD^+^-dependent deacetylases, is a key regulator of various processes and localized to the mitochondria matrix [10–12]. SIRT3 can regulate various processes through deacetylating its substrates at posttranslational modification [13–16]. Recently, some studies reported that SIRT3 blocks TGFβ1 signaling and aging-associated tissue fibrosis by deacetylating GSK3β [17]. In kidney, SIRT3 has been reported to protect cisplatin-induced acute kidney injury (AKI) [18, 19]. Moreover, AngII could downregulate SIRT3 mRNA expression in cultured tubular epithelial cells, and this effect might be prevented by an AT1 antagonist [20]. In addition, SIRT3 is upregulated by nicorandil and reduced by glibenclamide in dose-dependent manner in podocytes [15]. However, the function and possible molecular mechanism of SIRT3 in hypertensive nephropathy remains unclear.

Kruppel-like factors (KLFs) family includes 17 members and mediates cell growth and differentiation, metabolism and inflammation [21, 22]. KLF15 as a transcription factor, is widely expressed and enriched in kidney and liver [23, 24]. It has been reported that KLF15 regulates cardiac hypertrophy, adipogenesis, renal cells proliferation and differentiation [25, 26]. The previous report showed that KLF15 is required for restoring podocyte differentiation markers in mice and human podocytes under cell stress [27]. In addition, under high glucose, KLF15 could suppress mesangial cell proliferation and extracellular matrix (ECM) protein fibronectin expression via ERK1/2 MAPK signaling [28]. Moreover, previous evidence indicated that KLF15 is an essential negative regulatory factor for cardiac remodeling and renal interstitial fibrosis [22, 24, 29–31]. In cardiac fibroblasts, Transforming Growth Factor-β1 (TGFβ1) strongly reduces KLF15 expression, resulting in increased connective tissue growth factor (CTGF) [31]. Although KLF15 as a negative regulator of fibrosis contributes to the pathogenesis of various diseases, the molecule mechanism has not been fully understood. Whether SIRT3 could directly regulate and deacetylate KLF15 in hypertensive nephropathy requires further studies.

Honokiol (HKL), a low-molecular polyphenolic compound isolated from the bark of Magnolia officinalis (Houpo) [32–34], has been reported to activate SIRT3 and block cardiac hypertrophy [35]. What's more, HKL exhibits a variety of pharmacological activities, such as anti-inflammatory, antioxidant, antitumor and anti-thrombosis properties [36–40]. A previous study revealed that HKL inhibits tubulointerstitial fibrosis in rat UUO model by decreasing pro-inflammatory factors and pro-fibrotic factors [41]. In our study, we would explore whether HKL alleviate renal damage via elevating the expression of SIRT3 and activating its targets in hypertensive nephropathy.

Therefore, we were aimed to explore the role and possible molecular mechanism of SIRT3 in hypertensive nephropathy. Here, data from our study provided strong evidence that SIRT3 improved renal function and fibrosis by deacetylating KLF15. And for the first time, we identified that SIRT3-KLF15 signaling was involved in the development and progression of hypertensive nephropathy.

## RESULTS

### SIRT3 improved renal function in AngII-induced hypertensive nephropathy

**Figure 1:**
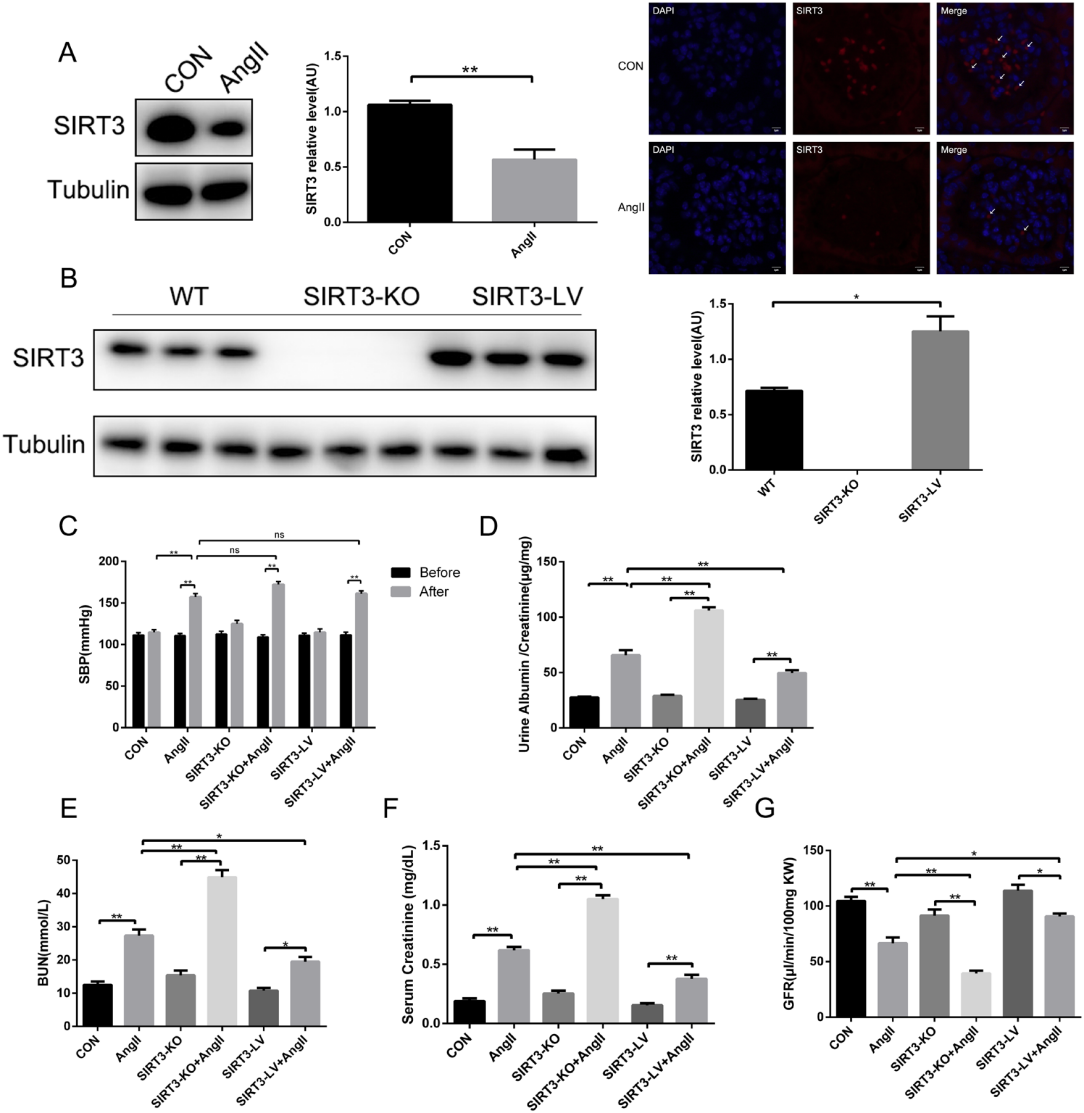
SIRT3 regulates renal function in hypertensive nephropathy **(A)** Representative Western blot analysis and quantification of SIRT3 with saline or AngII treatment in wild type mice. Representative immunofluorescence of SIRT3 (red) with saline or AngII treatment in murine kidney. DAPI stained nucleus in blue. Bars=5μm. (n=6) **(B)** Representative Western blot analysis and quantification of SIRT3 in WT, SIRT3-KO and SIRT3-LV mice (n=6). **(C)** Systolic blood pressure of six groups (n=6). **(D-G)** Ratio of urine creatinine to albumin, blood urea nitrogen, serum creatinine and glomerular filtration rate in WT, SIRT3-KO and SIRT3-LV mice infused with either saline or AngII for 42 days (n=6). The data are presented as the means ± SEM of three independent experiments. *P<0.05, **P<0.01.

To investigate the role of SIRT3 in hypertension-mediated kidney injury, WT mice, SIRT3-KO mice and SIRT3-LV mice were all subjected to AngII infusion for 42 days. Firstly, we confirmed SIRT3 expression in mouse kidneys and found that SIRT3 decreased in mice subjected to chronic AngII infusion compared with the controls infused with saline (Figure 1A and 1B). Secondly, blood pressure markedly increased in those subjected to AngII, but the changes affected by SIRT3 were of no statistical significance (Figure 1C). Thirdly, the ratio of urine creatinine to albumin, blood urea nitrogen (BUN) and serum creatinine (Scr) were all elevated in mice after AngII infusion, and especially in the SIRT3-KO group (Figure 1D-1F). Besides, the tendency of glomerular filtration rate (GFR) was opposite to those of urine creatinine to albumin, BUN and Scr (Figure 1G). Taken together, our findings showed that SIRT3-KO mice developed aggravated hypertensive nephropathy and deteriorated renal function, indicating that SIRT3 may be involved in the signaling pathways mediating hypertensive kidney injury.

### SIRT3 suppressed renal fibrosis in hypertensive nephropathy mice

**Figure 2:**
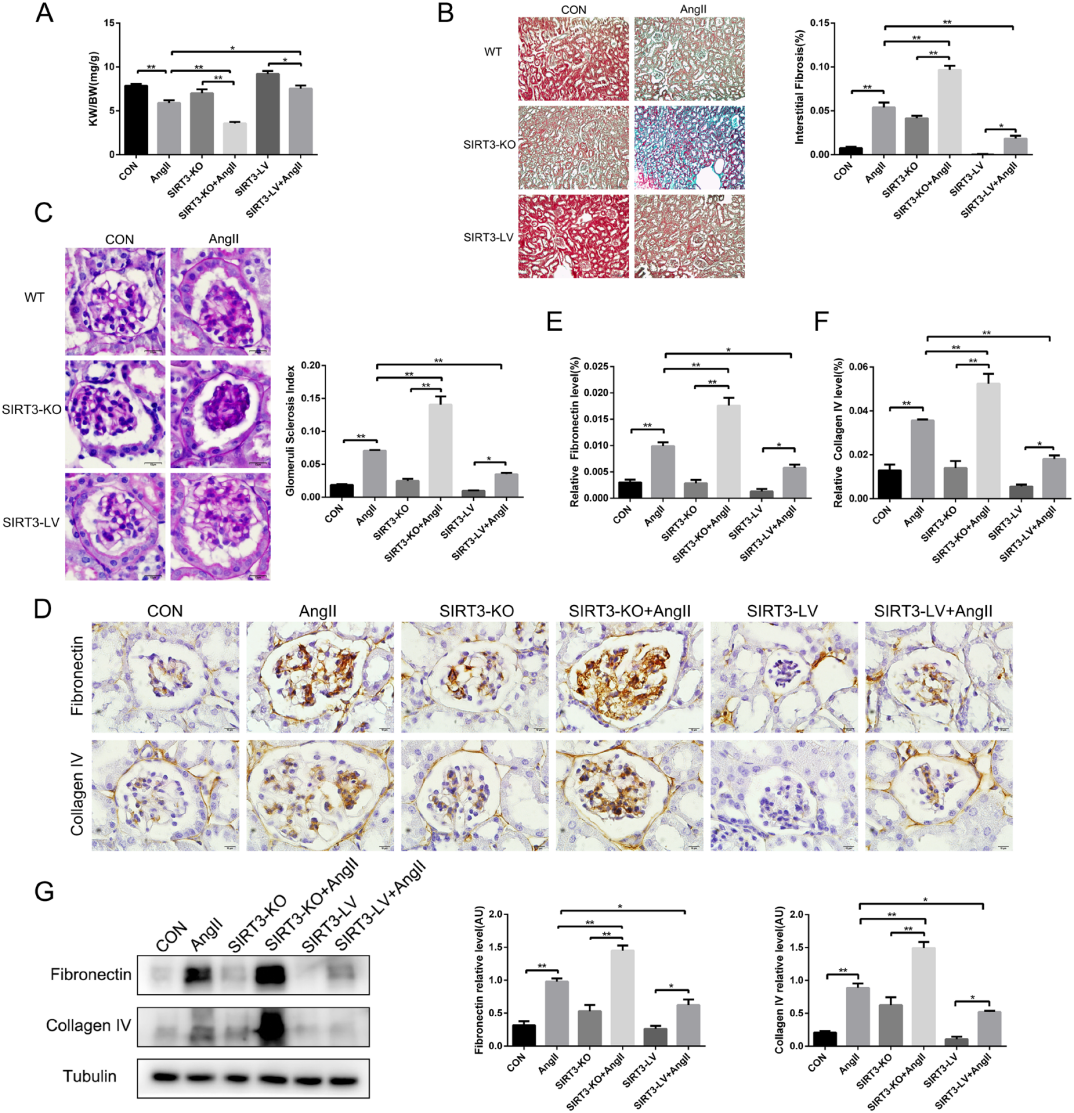
SIRT3 suppresses renal fibrosis *in vivo* **(A)** Ratio of the kidney weight to body weight in WT, SIRT3-KO and SIRT3-LV mice infused with either saline or AngII for 42 days (n=6). **(B)** Masson's trichrome staining showed the level of kidney fibrosis (green). Bars=50μm. The bar graph showing the quantification of kidney fibrosis (n=6). **(C)** Photomicrographs showing typical glomerular structure and quantification of glomerular sclerosis index in six different groups. Bars=10μm (n=6). **(D-F)** Immunohistochemistry analysis and quantification of fibronectin and collagen type IV in murine kidney. Bars=10μm (n=6). **(G)** Representative Western blot analysis and quantification of fibronectin and collagen type IV in murine kidney (n=6). The data are presented as the means ± SEM of three independent experiments. *P<0.05, **P<0.01.

Renal fibrosis is the key process of chronic kidney injury caused by hypertension. In order to preliminarily assess the kidney fibrosis, we weighed the kidney and body of each mouse in all groups at the end of the experiment and found that the ratio of kidney weight to body weight (KW/BW) decreased in AngII-infused mice, especially in the SIRT3-KO group (Figure 2A). The body weight and kidney weight were shown separately in Table 1. In addition, PAS and MASSON staining results showed that the areas of mesangial matrix and interstitial fibrosis increased in AngII-infused mice, of which the extent in SIRT3-KO mice was the greatest (Figure 2B and 2C). Furthermore, Western blot and immunohistochemistry confirmed that the expressions of fibronectin and collagen type IV increased in mice with chronic AngII treatment. And notably, SIRT3-LV mice exhibited lower level of fibrosis markers than WT and SIRT3-KO mice. (Figure 2D-2G). Altogether, SIRT3 may protect against renal fibrosis in hypertensive nephropathy.

**Table 1:** Body weight and kidney weight

	Control	AngII	SIRT3-KO	SIRT3-KO+AngII	SIRT3-LV	SIRT3-LV+AngII
**Body weight(g)**	24.92±0.37	25.24±0.52	25.20±0.40	25.09±0.46	24.34±0.33	24.70±0.33
**Kidney weight(mg)**	195.86±3.69	149.10±10.21*	176.58±8.91	89.45±3.88*#	223.67±6.55	186.59±10.37*#

Data are means± SEM from three independent experiments; n=6 mice/group.

*P<0.05, vs. the corresponding controls; #P<0.05, vs. the AngII group.

### SIRT3 prevented podocyte injury and decreased the expression of fibrosis factors in podocytes stimulated by AngII

**Figure 3:**
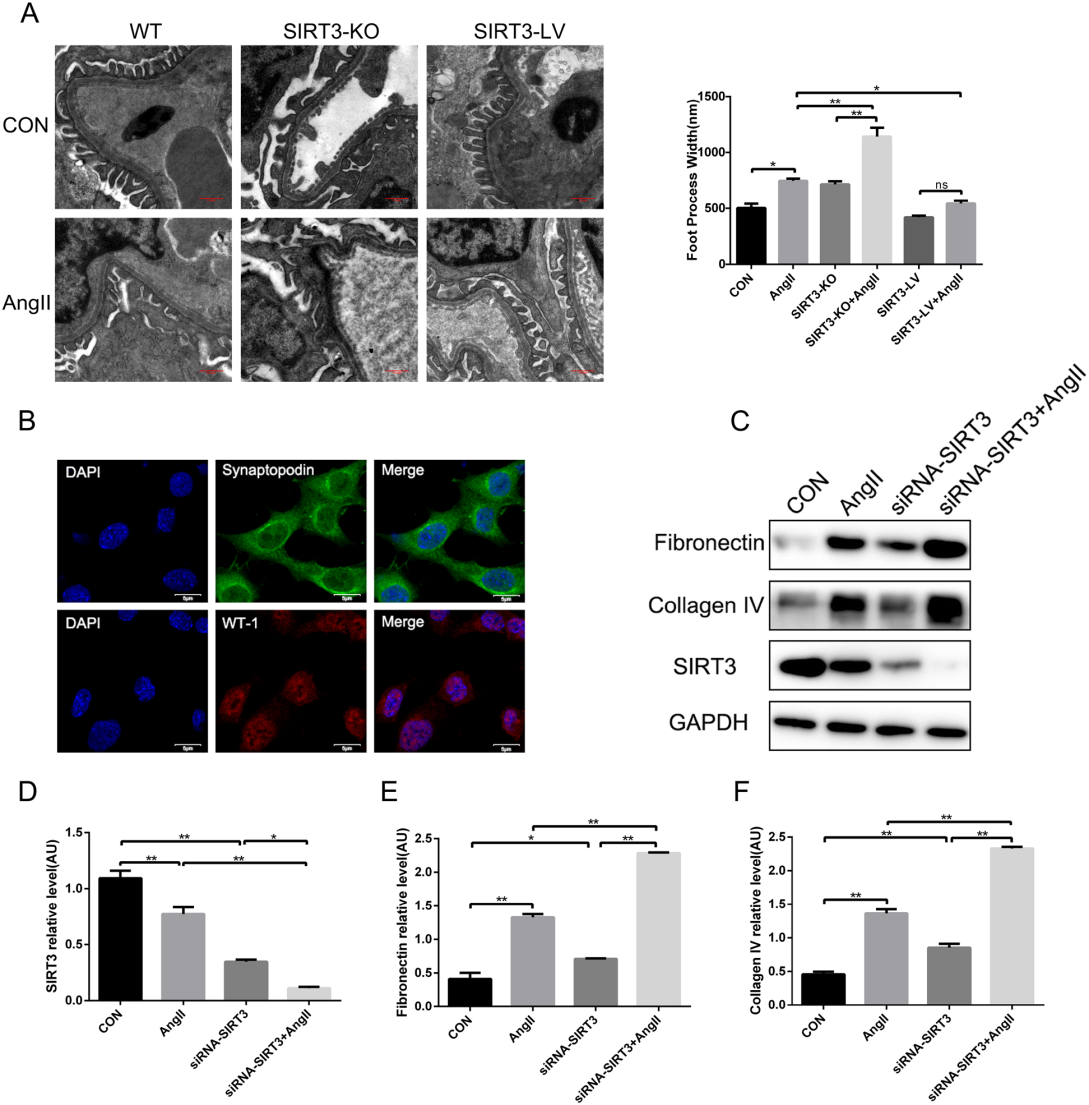
SIRT3 decreases the expression of fibrosis factors in podocytes **(A)** Morphological changes in the podocyte foot process by electron microscopy and quantification of foot process width. Bars=0.5μm (n=6). **(B)** Immunofluorescence of synaptopodin (green) and WT-1(red). DAPI stained nucleus in blue. Bars=5μm **(C-F)** Representative Western blot analysis and quantification of SIRT3, fibronectin and collagen type IV in MPC-5 podocytes. The data are presented as the means ± SEM of three independent experiments. *P<0.05, **P<0.01.

Podocytes are the important cells in glomerulus, which produce a number of fibrosis factors under damage. Firstly, we observed foot processes were fused or effaced in AngII-infused mice and SIRT3 ablation aggravated podocyte injury (Figure 3A). Secondly, in order to further investigate the effect of SIRT3 in cultured MPC-5 podocytes, we have verified the podocytes by the immunofluorescence of WT-1 and synaptopodin, which are the markers of podocytes (Figure 3B). And next, we knocked down SIRT3 through siRNA transfection in cultured MPC-5 podocytes and they were stimulated by AngII (10^−6^ mol/L, 48h). The results showed that the expressions of fibronectin and collagen type IV were elevated obviously with siRNA transfection and increased more following AngII stimulation (Figure 3C-3F). In sum, the above results revealed that SIRT3 could protect podocytes from damage and ameliorate the fibrosis phenotype of MPC-5 podocytes.

### SIRT3 interacted with KLF15 and deacetylated KLF15 *in vitro*

**Figure 4:**
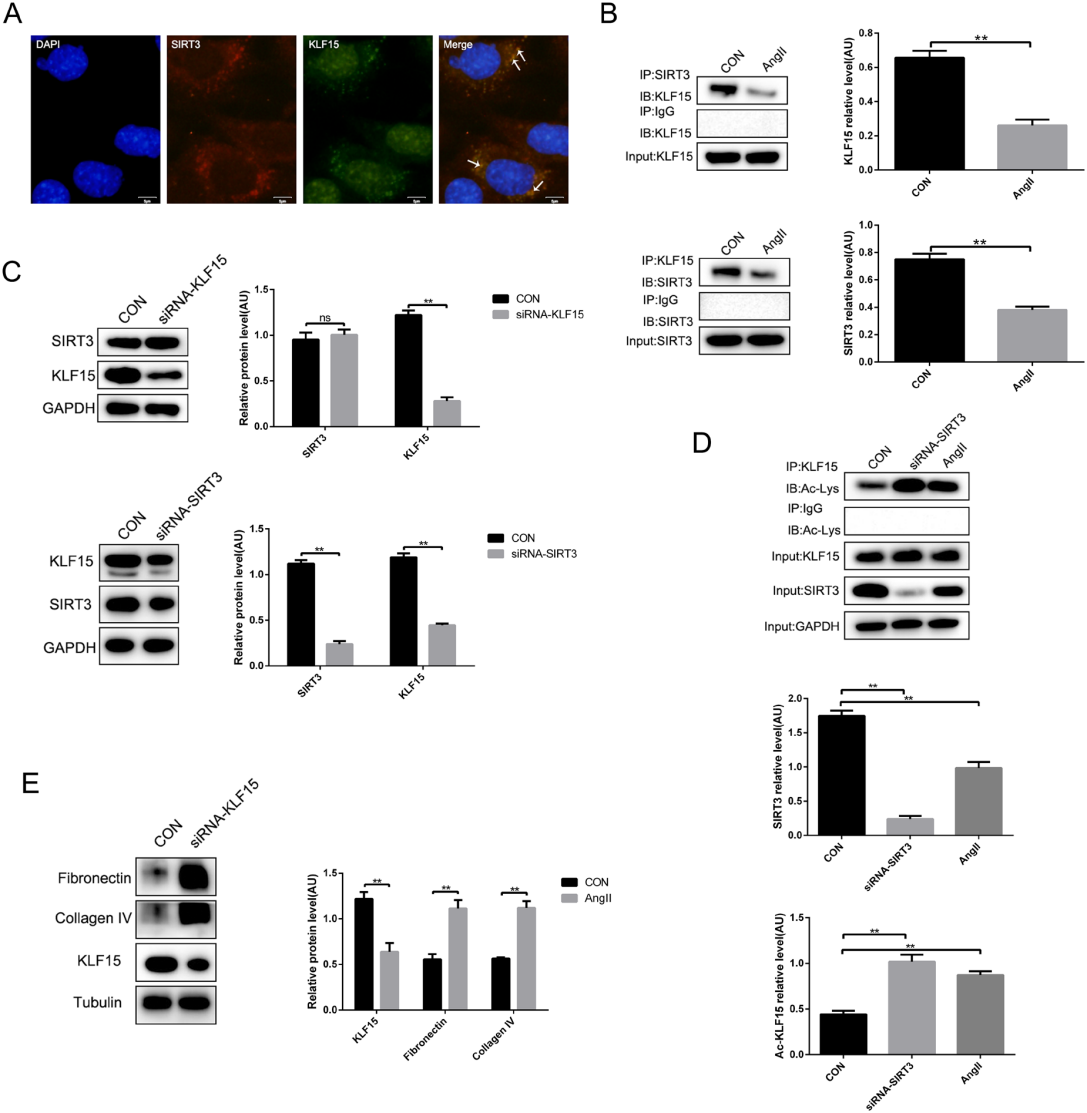
SIRT3 deacetylates KLF15 in podocytes **(A)** Immunofluorescence of SIRT3 (red) and KLF15 (green). DAPI stained nucleus in blue. Bars=5μm. **(B)** Co-immunoprecipitation analysis and quantification of the interaction between SIRT3 and KLF15 in MPC-5 cells. **(C)** Western blot analysis and quantification of SIRT3 and KLF15 after siRNA-KLF15 or siRNA-SIRT3 transfection *in vitro*. **(D)** Immunoprecipitation analysis and quantification of the KLF15 acetylation with SIRT3 knockdown or AngII stimulation in podocytes. **(E)** Western blot analysis and quantification of fibronectin and collagen type IV after KLF15 knockdown in MPC-5 cells. The data are presented as the means ± SEM of three independent experiments.*P<0.05, **P<0.01.

Previous evidence showed that KLF15 is an anti-fibrosis transcription factor. In this study, immunofluorescence showed a co-localization of SIRT3 and KLF15 in MPC-5 cells and they could interact directly with each other (Figure 4A and 4B). However, it was worth noting that the interaction between SIRT3 and KLF15 weakened after AngII infusion (Figure 4B). In addition, to make sure which are the upstream molecules of SIRT3 and KLF15, we measured the expression of them after siRNA transfection respectively in cultured MPC-5 cells by Western blot. The results showed that there was no change about the protein level of SIRT3 after KLF15 knockdown, whereas KLF15 expression was reduced following SIRT3 knockdown (Figure 4C). Therefore, we conjectured that KLF15 might be the target of SIRT3. Moreover, immunoprecipitation confirmed SIRT3 could deacetylate KLF15 at posttranslational modification in MPC-5 cells and the level of KLF15 acetylation was increased by AngII stimulation (Figure 4D). Furthermore, we knocked down KLF15 in MPC-5 cells, and found that the expressions of both fibronectin and collagen type IV were increased compared with the control cells (Figure 4E).

Altogether, it is suggested that the molecular mechanism of SIRT3 improving hypertensive renal fibrosis might be regulating the acetylation status of KLF15 in MPC-5 cells. Moreover, the decreased interaction of SIRT3 and KLF15 resulted in the reducing of KLF15 deacetylation by AngII stimulation.

### Honokiol alleviated hypertension-induced renal fibrosis by elevating the expression of SIRT3

**Figure 5:**
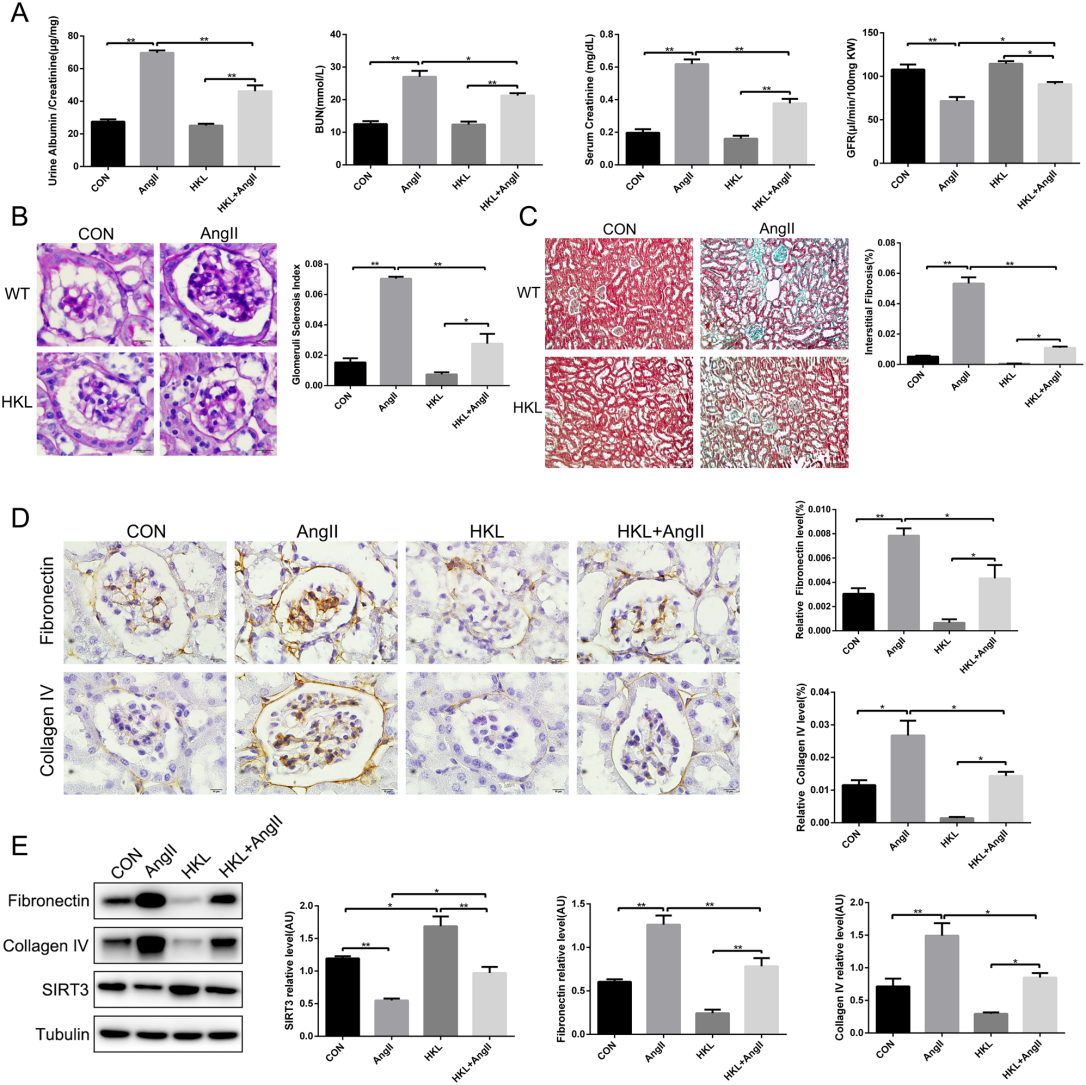
Honokiol alleviates hypertension-mediated kidney fibrosis by elevating SIRT3 **(A)** Ratio of urine creatinine to albumin, blood urea nitrogen, serum creatinine and glomerular filtration rate in control and AngII-infused mice with HKL treatment (n=6). **(B)** Photomicrographs showing typical glomerular structure and quantification of glomerular sclerosis index in four different groups. Bars=10μm (n=6). **(C)** Masson's trichrome stain and quantification of kidney fibrosis (green). Bars=50μm (n=6). **(D)** Immunohistochemistry analysis and quantification of fibronectin and collagen type IV in murine kidney with HKL treatment and AngII infusion. Bars=10μm (n=6). **(E)** Representative Western blot analysis and quantification of SIRT3, fibronectin and collagen type IV in kidney with HKL treatment and AngII infusion (n=6). The data are presented as the means ± SEM of three independent experiments.*P<0.05, **P<0.01.

To investigate the effect of honokiol in hypertension-mediated kidney injury, both AngII-infused WT mice and their controls were subjected to honokiol for 42 days by intraperitoneal injection. Firstly, renal function was improved by HKL treatment (Figure 5A). Secondly, PAS stain and MASSON stain showed that HKL decreased the extent of glomerulosclerosis and kidney fibrosis (Figure 5B and 5C). Thirdly, we found elevated SIRT3 expression and the decreased fibronectin and collagen type IV after HKL treatment. (Figure 5D and 5E). These findings revealed that HKL might alleviate hypertension-induced renal fibrosis through elevating SIRT3 expression.

### Honokiol activated SIRT3-KLF15 signaling and protected podocytes against fibrosis by AngII infusion

**Figure 6:**
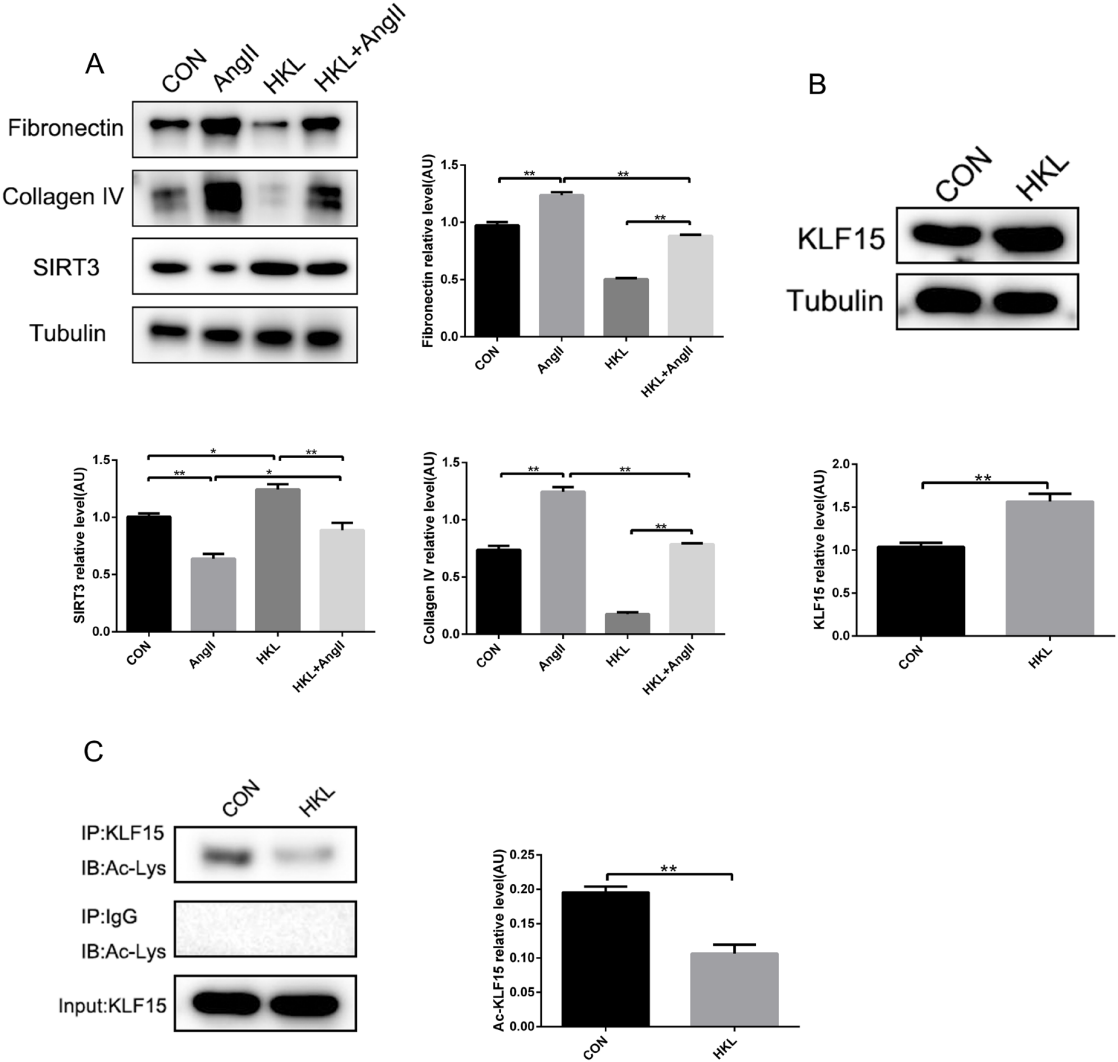
Honokiol activates SIRT3-KLF15 signaling **(A)** Representative Western blot analysis and quantification of SIRT3, fibronectin and collagen type IV in MPC-5 cells with HKL treatment and AngII infusion. **(B)** Representative Western blot analysis and quantification of KLF15 with HKL treatment in MPC-5 cells. **(C)** Immunoprecipitation analysis and quantification of the KLF15 acetylation with HKL treatment in podocytes. The data are presented as the means ± SEM of three independent experiments.*P<0.05, **P<0.01.

*In vitro*, MPC-5 cells were incubated with AngII (10^−6^ mol/L, 48h) following HKL stimulation (10μM, 1h). The results showed HKL treatment decreased fibrosis factors but increased SIRT3 expression (Figure 6A). What's more, to further validate the activated status of SIRT3-KLF15 signaling affected by HKL, immunoprecipitation and Western blot confirmed, that the expression and the deacetylation status of KLF15 were elevated by HKL treatment (Figure 6B and 6C). These results suggested that HKL can alleviate fibrosis via activating the SIRT3-KLF15 signaling in podocytes.

**Figure 7:**
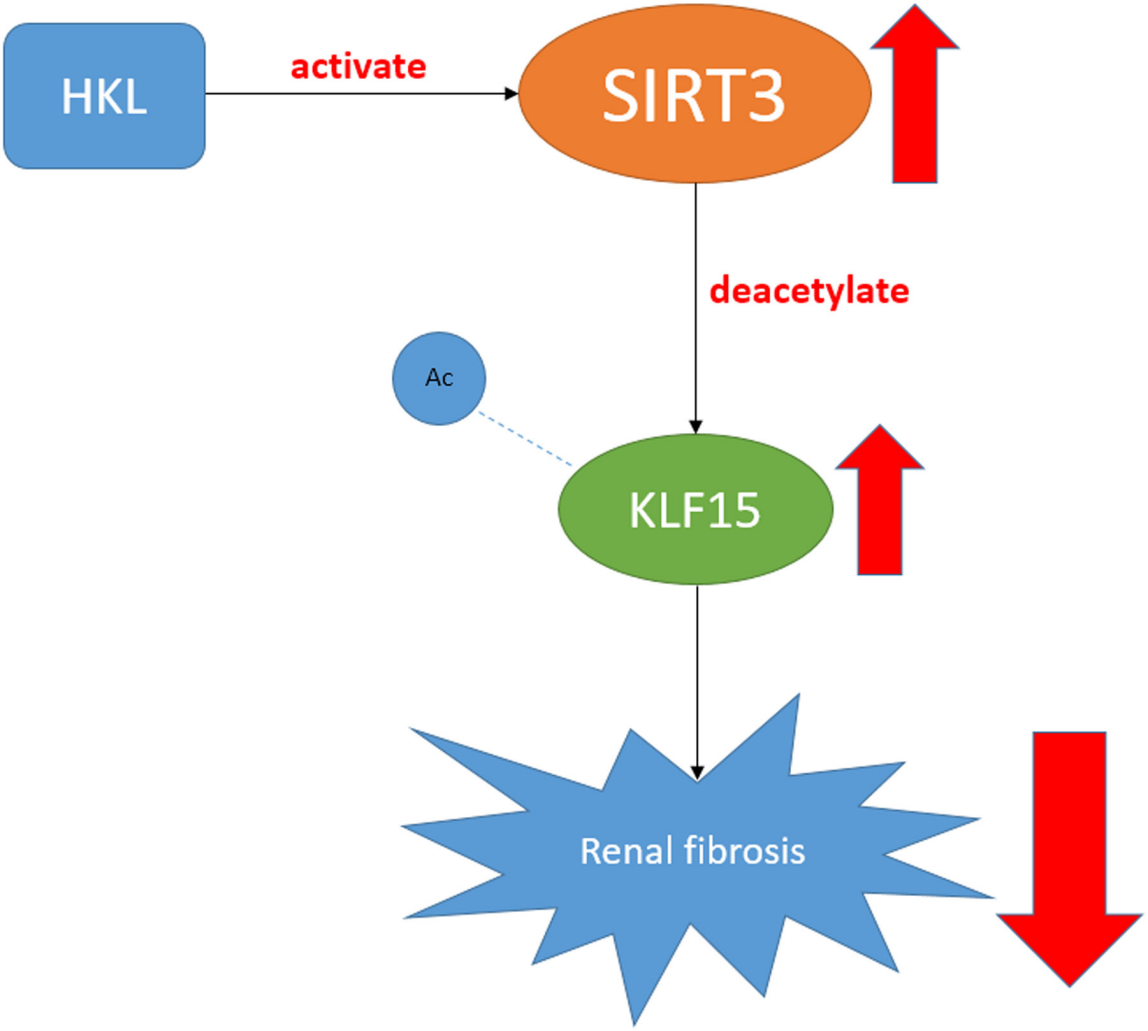
A figure shows the SIRT3-KLF15 signaling pathway

Taken together, the findings revealed that SIRT3 ameliorated renal function and protected against renal fibrosis in AngII-induced hypertensive nephropathy. What's important is that SIRT3 might deacetylate KLF15 and activate its anti-fibrosis function in podocytes. In addition, HKL treatment alleviated kidney injury via activating the SIRT3-KLF15 signaling (Figure 7).

## DISCUSSION

Renal fibrosis involves in the process of hypertension. The recent studies mainly focused on the effect of SIRT3 in acute kidney injury and diabetic nephropathy. In this study, we studied the function and molecular mechanism of SIRT3 in hypertensive nephropathy. First of all, on account of the apoptosis of kidney inherent cells, glomerulus and kidney tubules atrophy in the progression of renal fibrosis, the kidney weight of SIRT3-KO hypertensive mice reduced at a largest extent as shown in Table 1. It is suggested that SIRT3 may play a protective role in hypertensive kidney injury. Moreover, SIRT3 ablation aggravated renal function impairment and renal fibrosis in hypertensive nephropathy. However, SBP was not affected by SIRT3. It reveals that SIRT3 may directly regulate its targets rather than changing the blood pressure to prevent kidney injury. Therefore, we further investigated the targets and molecular mechanism *in vitro*.

Podocytes are an important part of the glomerular filtration membrane in kidney and its foot processes interlinked by slit diaphragms serve to prevent the filtration of protein from the blood [42–45]. Podocyte injury is associated with proteinuria and consequent kidney fibrosis that progresses to chronic kidney disease [46–48]. Therefore, we made a preliminary observation of the morphological changes in podocyte foot process in murine kidney. We found that foot processes were fused or effaced in AngII-infused mice and the changes were even worse in SIRT3-KO group. It is suggested that SIRT3 may prevent podocyte damage in hypertensive nephropathy. Next, we further investigated the target and molecular mechanism of SIRT3 involved in renoprotective effect in podocytes. For one thing, we found that SIRT3 could bound to KLF15 in cultured MPC-5 cells. Moreover, SIRT3, as a NAD+-dependent deacetylase, also regulated the acetylation status of KLF15 at posttranslational modification. In addition, we observed a co-localization of SIRT3 and KLF15 in the cytoplasm. Taken together, we speculate that SIRT3 may deacetylate KLF15 in the cytoplasm and the deacetylated KLF15 is translocated to nuclear to activate its anti-fibrosis function. For another, the results showed that the interaction between SIRT3 and KLF15 reduced and the acetylated KLF15 increased by AngII stimulation. However, we didn't investigate whether the co-localization of SIRT3 and KLF15 decreased with AngII stimulation. In a word, we concluded that the SIRT3-KLF15 signaling activation suppressed renal fibrosis and ameliorated renal function in hypertensive nephropathy. To our knowledge, this study is the first to describe a role of SIRT3 in hypertensive nephropathy via deacetylating KLF15.

We also investigated HKL effect in AngII-induced kidney injury. The results showed that HKL increased SIRT3 level and improved AngII-induced renal damage *in vivo* and *in vitro*. In addition, we further observed that HKL not only upregulated the expression of KLF15 but also increased the KLF15 deacetylation. It indicated that HKL might serve as a SIRT3-KLF15 signaling agonist to prevent hypertensive nephropathy.

In conclusion, for the first time, our findings demonstrate that SIRT3 plays a renoprotective role by deacetylating KLF15 and the SIRT3-KLF15 signaling may be a novel pathway to contribute to prevent hypertensive nephropathy. What's more, it is revealed that honokiol alleviates hypertension-induced renal damage as a SIRT3-KLF15 signaling agonist. HKL might provide a new approach to combating renal damage and fibrosis in hypertensive nephropathy.

## MATERIALS AND METHODS

### Ethics statement

The animal experimental protocol and animal care procedures complied with the Animal Management Rules of the Ministry of Public Health, People's Republic of China (documentation No 55, 2001) and were approved by the Animal Care Committee of Shandong University.

### Materials and reagents

Mini-osmotic pumps were purchased from DURECT Corporation (Model 2006, Cupertino, CA). Angiotensin II (AngII), Honokiol (HKL), FITC-inulin, Bovine Serum Albumin (BSA) were purchased from Sigma Aldrich (Sigma Aldrich, USA). Mouse urine creatinine assay kit was purchased from R&D Systems (R&D, USA). Mouse urine albumin, BUN and Scr assay kit were purchased from AssayPro Corporation (AssayPro, USA). Antibodies against SIRT3 (28kDa), acetylated lysine, Tubulin and GAPDH were purchased from Cell Signaling Technology (CST, USA). Antibodies against KLF15, fibronectin, collagen type IV, synaptopodin, WT-1 were purchased from abcam (Abcam, USA).

### Animal model

129 wild-type (WT) male mice of 8 weeks old were purchased from Department of Laboratory Animal Science of Peking University (Beijing, China) and SIRT3-knockout (SIRT3-KO) mice were purchased from the Jackson Laboratories (USA). SIRT3-overexpression (SIRT3-LV) mice were obtained by injection to the 129 WT mice SIRT3-overexpression-lentivirus (JIKAI GENE, Shanghai, China) via caudal vein. HKL were intraperitoneally injected in 129 WT mice for 42 days (25 mg/Kg, once a day) as the HKL treatment group. All experimental mice anesthetized with isoflurane (1%) were implanted subcutaneously corresponding osmotic pumps beforehand filled with AngII or saline for 42 days. The osmotic pumps infused with appropriate doses of AngII in sterile saline (2000 ng/kg per min) or saline according to the directions were placed in sterile 0.9% saline at 37°C for 60 hours to prime. Together, the experimental animals were randomly assigned to eight groups: WT+saline group, WT+AngII group, SIRT3-KO+saline group, SIRT3-KO+AngII group, SIRT3-LV+saline group, SIRT3-LV+AngII group, WT+HKL+saline group, WT+HKL+AngII group. Mice were sacrificed at the end of the experiment and the kidneys were used to assay for renal fibrosis. In addition, Blood pressure were measured by Tail-Cuff way (BP2010A, softron, Japan) before modeling and death.

### Renal function

Urine albumin and creatinine, BUN, serum creatinine were detected by the ELISSA kit according to the direction of the kit and GFR was measured as previously described using fluorescein isothiocyanate label inulin (FITC-inulin, Sigma) [49].

### Transmission electron microscopy

Mice were perfused with PBS and then immediately fixed in 2.5% glutaraldehyde for electron microscopy as previously described and the images were observed using a transmission electron microscope (TEM, H-7000FA, Hitachi, Tokyo, Japan) [27, 50]. Podocyte effacement was quantified as previously described [51].

### Histology and immunohistochemistry

Periodic acid Schiff (PAS) staining and MASSON trichrome staining were performed to evaluate the level of glomerulosclerosis and renal fibrosis. The glomerulosclerotic index and collagen volume fraction was evaluated blindly by an automated image analysis system (Image-Pro Plus, Version 6.0, USA). The antibodies anti-fibronectin (1:200 dilution) and anti-collagen type IV (1:400 dilution) were used to immunohistochemical staining and photomicrographs were quantified also using Image-Pro Plus 6.0.

### Cell culture

Conditionally immortalized mouse podocytes (MPC-5) were donated by Dr. Peter Mundel of Harvard Medical School (Boston, MA), and cultured with DMEM (Gibco) containing 10% fetal bovine serum (FBS, Gibco) at 33°C to propagate. To induce differentiation, podocytes were incubated at 37°C for 14 days and used for experiments in this study.

### Immunofluorescence

To identify podocytes, immunofluorescence assay with synaptopodin or WT-1 acted as the marker protein of differentiation maturation podocytes was necessary. Cultured podocytes were fixed with 4% paraformaldehyde, treated with 0.5% Triton X–100, blocked with 1%BSA and then incubated with antibodies anti- synaptopodin or anti- WT-1. All imaging analyses were used in laser scanning confocal microscope after the incubating with the secondary antibodies.

### Transfection and treatment

To knockdown SIRT3 or KLF15 *in vitro*, the podocytes were transfected with 50 nM of small interfering RNA (siRNA) with Lipofectamine 2000 (Invitrogen) for 48h. In addition, podocytes were stimulated by AngII (10^−6^M) for 48h following HKL (10μM) treatment 1h.

### Western blot

Cultured cells or isolated kidney cortex were lysed by the addition of lysis buffer containing 50 mM Tris-buffer (pH 7.4), 150 mM NaCl, 0.5 mM EDTA, 0.5% NP40, and 1% Triton X-100, Phenylmethanesulfonyl fluoride. Protein samples were subjected to electrophorese on 10% SDS/PAGE gels before the disconnected proteins transferring to PVDF membrane in a designated time and then blocked with 5% skim milk in 1X TBST for 2h. The blocked membrane were incubated with corresponding antibodies at 4°C overnight. The membrane were washed with 1X TBST in the second day and then incubated with the secondary antibody in 1:5000 dilution. After washing with 1X TBST, the protein band were showed by enhanced chemiluminescence (Millipore) and an Image Quant LAS4000 chemiluminescence reader (GE, USA).

### Immunoprecipitation

200μg proteins of cell lysates were used with Protein A+G Agarose and IgG to remove nonspecific binding according to manufacturer's instructions. SIRT3 or KLF15 was immunoprecipitated with their corresponding antibodies and then the samples were performed immunoblotting according to the above.

### Statistical analysis

Data were shown as mean ± SEM. Statistical analysis was performed with GraphPad Prism 6 (La Jolla, CA, USA) and using unpaired t test to analyze data between two groups. To assess differences among groups, ANOVA followed by Tukey post-hoc test were used. P<0.05 was considered significant.
